# SerpinA3 is an Endogenous TGF‐β Receptor Antagonist that Attenuates Cardiac Fibroblast Activation and Fibrotic Remodeling

**DOI:** 10.1002/advs.77379

**Published:** 2026-08-30

**Authors:** Hui Wang, Jieyu Cui, Chunlu Huang, Wanyi Guo, Tong Liu, Yi Zhu, Shuai Shao, Kangyin Chen, Jinlong He, Qiankun Bao

**Affiliations:** ^1^ Tianjin Key Laboratory of Ionic‐Molecular Function of Cardiovascular Disease Department of Cardiology Tianjin Institute of Cardiology the Second Hospital of Tianjin Medical University Tianjin China; ^2^ NHC Key Laboratory of Hormones and Development Province and Ministry Co‐Sponsored Collaborative Innovation Center for Medical Epigenetics Department of Physiology and Pathophysiology Tianjin Medical University Tianjin China

**Keywords:** cardiac fibroblast, cardiac fibrosis, SerpinA3, TGF‐β receptor, TGF‐β signaling pathway

## Abstract

**Background**: Cardiac fibrosis is a central driver of adverse remodeling and heart failure (HF), yet effective antifibrotic therapies remain limited. SerpinA3, a member of the serine protease inhibitor family, has been implicated in cardiovascular disease, but its functional role and underlying mechanisms in cardiac remodeling are poorly defined.

**Methods and Results**: Using a transverse aortic constriction (TAC) model, we observed that SerpinA3K expression was markedly reduced in failing mouse hearts and in circulation. Pharmacological supplementation or genetic augmentation of SerpinA3 markedly attenuated cardiac dysfunction and fibrotic remodeling in response to pressure overload. Single‐cell transcriptomic analyses further demonstrated that SerpinA3 suppresses profibrotic fibroblast programs by promoting a transition from activated and extracellular matrix–producing fibroblasts toward a quiescent, pro‐angiogenesis state. Mechanistically, SerpinA3 binds to the extracellular domain of TGF‐beta receptor type‐1, and disrupts TGF‐β receptor type‐1–TGF‐β receptor type‐2 complex formation, thereby preventing downstream Smad2/3 phosphorylation in cardiac fibroblasts.

**Conclusions**: SerpinA3 functions as an endogenous inhibitor of TGF‐β signaling that restrains cardiac fibroblast activation and fibrotic remodeling. By targeting receptor complex assembly, SerpinA3 provides a selective mechanism to modulate profibrotic signaling. These findings identify SerpinA3 as a promising therapeutic target for HF associated with pathological fibrosis.

## Graphical abstract

SerpinA3 acts as an endogenous TGF‐β receptor antagonist that binds to the extracellular domain of TGFR‐1, thereby preventing TGFR‐1–TGFR‐2 complex formation. This receptor‐level blockade suppresses TGF‐β/Smad2/3 signaling, attenuates cardiac fibroblast activation, and extracellular matrix deposition, and ultimately alleviates pressure overload–induced cardiac fibrosis.

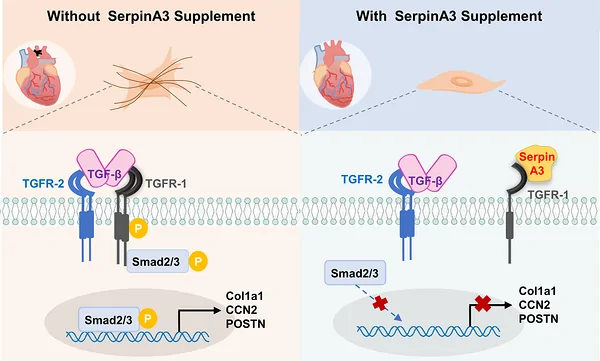

## 1 Introduction

Heart failure (HF) is a leading cause of morbidity and mortality worldwide and results from structural, functional, electrical, or conduction abnormalities of the heart [1, 2]. Among the pathological processes driving HF, cardiac fibrosis represents a central and largely irreversible feature, characterized by excessive activation of cardiac interstitial cells and aberrant deposition of extracellular matrix (ECM) [3]. Progressive fibrotic remodeling promotes left ventricular stiffening and dilation, ultimately impairing both diastolic and systolic function [4]. Despite its fundamental contribution to HF progression, effective anti‐fibrotic therapies remain limited, underscoring an unmet clinical need.

Serpins (serine protease inhibitors) constitute a large and evolutionarily conserved superfamily of proteins characterized by a shared tertiary structure and originally defined by their ability to inhibit serine proteases [5]. In humans, the Serpin family comprises 36 protein‐coding genes and five pseudogenes, whereas the mouse genome encodes approximately 60 functional Serpin genes, many of which are orthologous to human Serpins, with others expanded into species‐specific paralogous clusters [6]. Among them, SerpinE1, also known as plasminogen activator inhibitor‐1 (PAI‐1), is predominantly produced by endothelial cells and serves as a key regulator of fibrinolysis [7]. Elevated circulating PAI‐1 levels are strongly associated with increased risk of thrombosis and atherosclerosis [7, 8]. Although, the biologic functions of human serpins involved in the clotting and fibrinolytic cascades are well documented, however, the role of human serpins in biologic processes in cardiac remodeling and heart failure awaits further validation.

Transforming growth factor‐β (TGF‐β) signaling is a master regulator of fibroblast activation and cardiac fibrosis [9]. Activated TGF‐β binding to the TGF‐beta receptor type‐2 (TGFR‐2) induces recruitment and phosphorylation of TGF‐beta receptor type‐1 (TGFR‐1), leading to activation of Smad2/3‐dependent transcriptional programs [10]. Although pharmacological inhibition of TGF‐β or TGFR‐1 has shown anti‐fibrotic efficacy in experimental models, clinical translation has been hampered by dose‐limiting cardiotoxicity [11]. Alternative strategies aimed at disrupting ligand–receptor interactions at the extracellular domain have been explored, yet no approved therapies have emerged.

Here, we demonstrate that SerpinA3 protein levels are markedly reduced in both cardiac tissue and plasma of mice subjected to transverse aortic constriction (TAC). Pharmacological or genetic methods to increase SerpinA3 levels attenuated pressure overload‐induced cardiac dysfunction and fibrosis. Single‐cell transcriptomic analysis identified SerpinA3 as an endogenous suppressor of TGF‐β signaling in cardiac fibroblasts, thereby limiting fibroblast activation. Mechanistically, SerpinA3 directly interacts with TGFR‐1 and interferes with the formation of the TGFR‐1–TGFR‐2 signaling complex. Collectively, our findings identify SerpinA3 as a critical regulator of cardiac fibroblast activation and pressure overload–induced cardiac remodeling, highlighting its therapeutic potential in HF.

## 2 Results

### 2.1 SerpinA3K Expression is Decreased in Failing Heart

TAC was used to induce pressure overload–mediated cardiac remodeling and heart failure in mice [12, 13]. 8 weeks after TAC, mice exhibited marked cardiac dysfunction, and hypertrophic remodeling compared with sham controls, as evidenced by reduced ejection fraction (EF), and fractional shortening (FS), along with increased interventricular septal thickness in diastole (IVSTd), left ventricular posterior wall thickness in diastole (LVPWd), left ventricular internal dimensions in systole and diastole (LVIDs and LVIDd), and left ventricular (LV) mass (Figure S1A–G).

**FIGURE 1:**
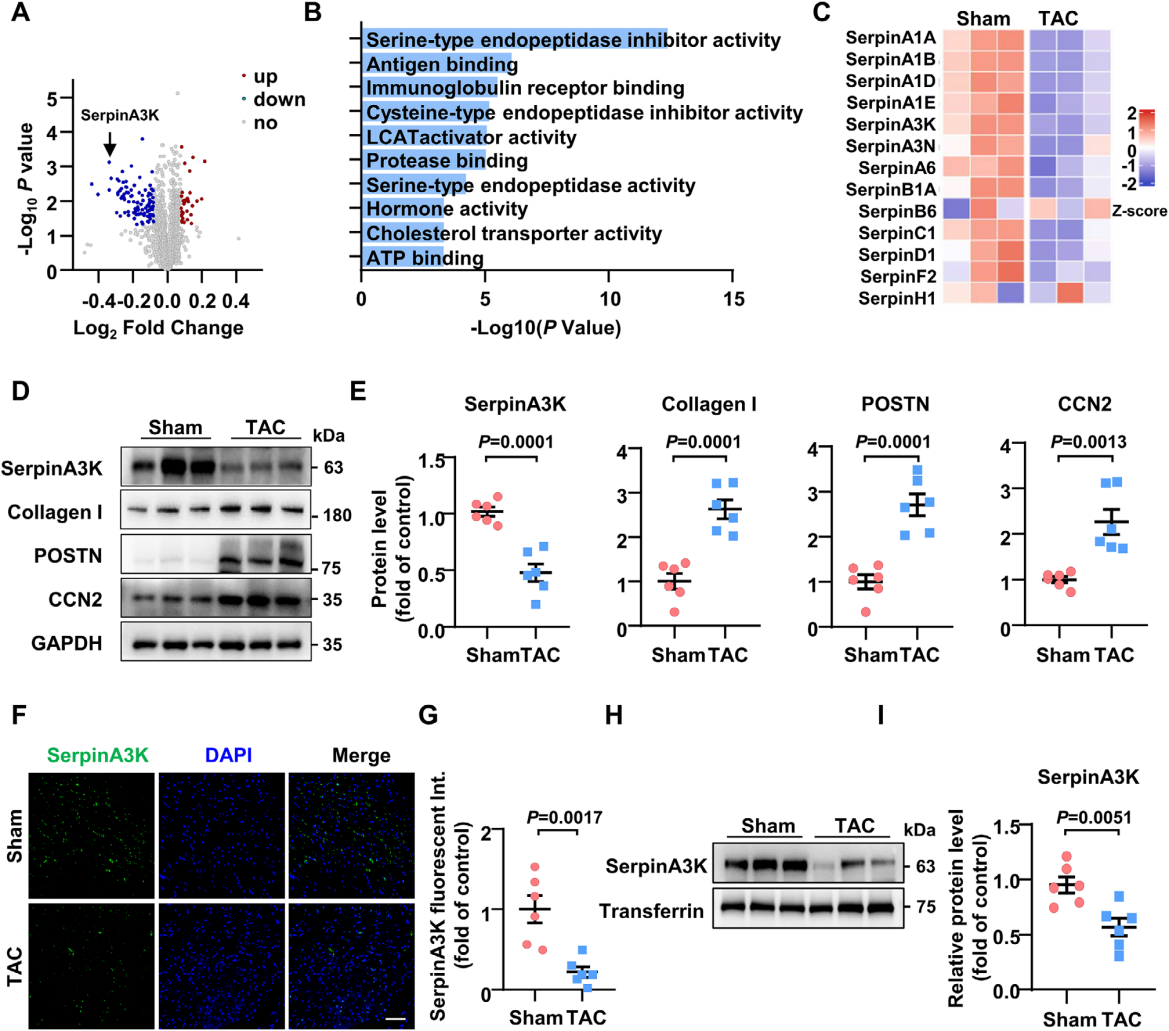
SerpinA3K is downregulated in the pressure overload–induced failing heart. C57BL/6J mice underwent TAC surgery for 8 weeks; the control group underwent Sham surgery. (A) Left ventricular tissues from Sham and TAC mice were analyzed by tandem mass tag‐based quantitative proteomic analysis (*n* = 3). The volcano map shows differentially expressed proteins between the 2 groups. (B) Gene Ontology analysis of the differentially expressed genes between the 2 groups. (C) The heatmap illustrates the expression differences of proteins related to serine‐type endopeptidase inhibitor activity between the 2 groups. (D) Protein levels of SerpinA3K, Collagen I, Periostin (POSTN), and Connective tissue growth factor (CCN2) in the left ventricle of mice detected by western blot analysis. (E) Quantification of SerpinA3K, Collagen I, POSTN, and CCN2 in (D). (F) Representative confocal microscopy images of immunofluorescence staining for SerpinA3K (green) and DAPI (blue) of the left ventricle. Scale bar: 50 µm. (G) Quantification of SerpinA3K fluorescence intensity in (F). (H) Protein levels of SerpinA3K in plasma of mice detected by western blot analysis. (I) Quantification of SerpinA3K in (H). Data are mean ± SEM, *n* = 6 mice per group, unpaired 2‐tail *t* test.

To identify proteins potentially involved in pressure overload–induced heart failure, we performed tandem mass tag (TMT)–based quantitative proteomic profiling of left ventricular tissue from sham‐ and TAC‐operated C57BL/6J mice. Principal component analysis (PCA) score plot showed that the biological replicates within each group clustered closely and the protein expression profile significantly changed after TAC operation (Figure S1H). A total of 130 proteins were differentially expressed following TAC, including 33 upregulated and 97 downregulated proteins (Figure 1A). Gene Ontology analysis revealed significant enrichment of proteins associated with serine‐type protease inhibitor activity (GO:0004867) among the differentially expressed proteins (Figure 1B). Within this category, clustering analysis identified SerpinA3K, which is the functional ortholog to human SerpinA3, as the most prominently downregulated protein in TAC‐induced failing hearts (Figure 1C and Figure S1I and Table S1).

Consistent with the proteomic findings, immunoblot analysis confirmed a marked reduction of SerpinA3K protein levels in TAC hearts, concomitant with increased expression of fibrotic markers including collagen I, periostin (POSTN), and connective tissue growth factor (CTGF/CCN2), compared with sham controls (Figure 1D,E). Immunofluorescence staining further demonstrated a robust decrease in SerpinA3K expression in failing myocardium following TAC (Figure 1F,G). Given that SerpinA3K is a secreted protein, we next assessed its circulating levels and observed a significant reduction in plasma SerpinA3K in TAC mice relative to sham mice (Figure 1H,I). To determine the cellular distribution of SerpinA3K in the heart, we performed immunofluorescence co‐staining with markers for fibroblasts (vimentin), myofibroblasts (α‐SMA), endothelial cells (CD31), and cardiomyocytes (α‐actinin) in left ventricular tissue. SerpinA3K was predominantly expressed in fibroblasts, myofibroblasts, and endothelial cells. Notably, TAC‐induced pressure overload markedly reduced SerpinA3K expression in fibroblasts and myofibroblasts, whereas its expression in endothelial cells remained largely unchanged (Figure S1J,K).

### 2.2 SerpinA3 Ameliorates Pressure Overload–Induced Cardiac Dysfunction and Remodeling

**FIGURE 2:**
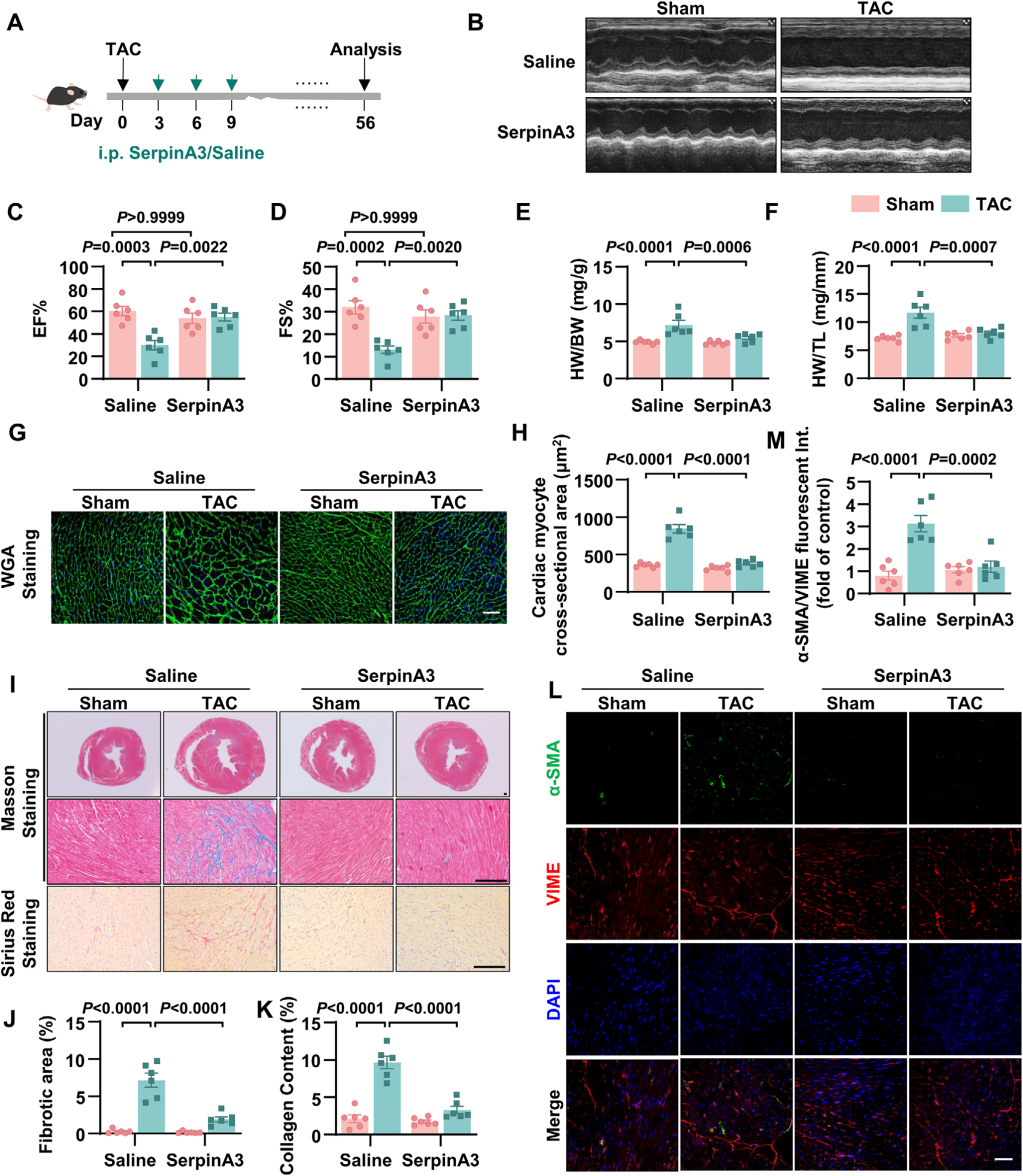
SerpinA3 attenuates pressure overload–induced cardiac dysfunction and adverse remodeling. (A) C57BL/6J mice underwent Sham or TAC surgery, followed by intraperitoneal injection of recombinant SerpinA3 protein (100ug/kg) every 3 days for 8 weeks. The control group was injected with sterile saline. (B) M‐mode echocardiographic images of 4 groups mice. (C, D) Echocardiographic analysis of left ventricular ejection fraction (EF) and fractional shortening (FS) in each group. (E, F) Ratio of heart weight (HW) to body weight (BW), and ratio of heart weight (HW) to tibial length (TL) in each group. (G) Representative images of Gated wheat germ agglutinin (WGA) staining of the left ventricle. Scale bar: 50 µm. (H) Quantification of the cross‐sectional area of the left ventricular cardiomyocytes in (G). (I) Representative images of Masson staining and Sirius red staining of the left ventricle. Scale bar: 200 µm. (J, K) Quantification of fibrotic area and collagen content of the left ventricular in (I). (L) Representative confocal microscopy images of immunofluorescence staining for a‐SMA (green) and vimentin (VIME, fibroblast marker) (red). Scale bar: 50 µm. (M) Quantification of co‐localization of a‐SMA and VIME fluorescence intensity in (L). Data are mean ± SEM, *n* = 6 mice per group, 2‐way ANOVA with Bonferroni post‐test.

We next examined whether exogenous SerpinA3 could mitigate pressure overload–induced cardiac dysfunction and remodeling. Mice were subjected to TAC and administered recombinant human SerpinA3 via intraperitoneal injection (Figure 2A). 8 weeks after surgery, SerpinA3 treatment significantly improved cardiac systolic function, as evidenced by restoration of EF and FS, and attenuated TAC‐induced increases in IVSTd, LVPWd, LVIDs, LVIDd, and LV mass (Figure 2B–D and Figure S2A–E).

Consistently, SerpinA3‐treated TAC mice exhibited significantly reduced heart weight–to–body weight (HW/BW) and heart weight–to–tibia length (HW/TL) ratios compared with vehicle‐treated TAC controls (Figure 2E,F). Wheat germ agglutinin (WGA) staining demonstrated that SerpinA3 markedly attenuated TAC‐induced cardiomyocyte hypertrophy (Figure 2G,H). Furthermore, Masson's trichrome and Sirius Red staining revealed a substantial reduction in myocardial fibrotic area and collagen deposition following SerpinA3 treatment (Figure 2I–K).

At the cellular level, immunofluorescence analysis showed that SerpinA3 treatment significantly suppressed TAC‐induced α‐smooth muscle actin (α‐SMA) expression in vimentin‐positive cardiac fibroblasts (CFs), indicating reduced fibroblast activation (Figure 2L,M). Collectively, these results demonstrate that SerpinA3 administration preserves cardiac function and limits cardiac fibroblast activation and pathological remodeling in response to pressure overload. These beneficial effects on cardiac remodeling were accompanied by improved survival in SerpinA3‐treated mice compared with vehicle‐treated TAC controls (Figure S2F).

### 2.3 SerpinA3 Suppresses Cardiac Fibroblast Activation in Failing Hearts

**FIGURE 3:**
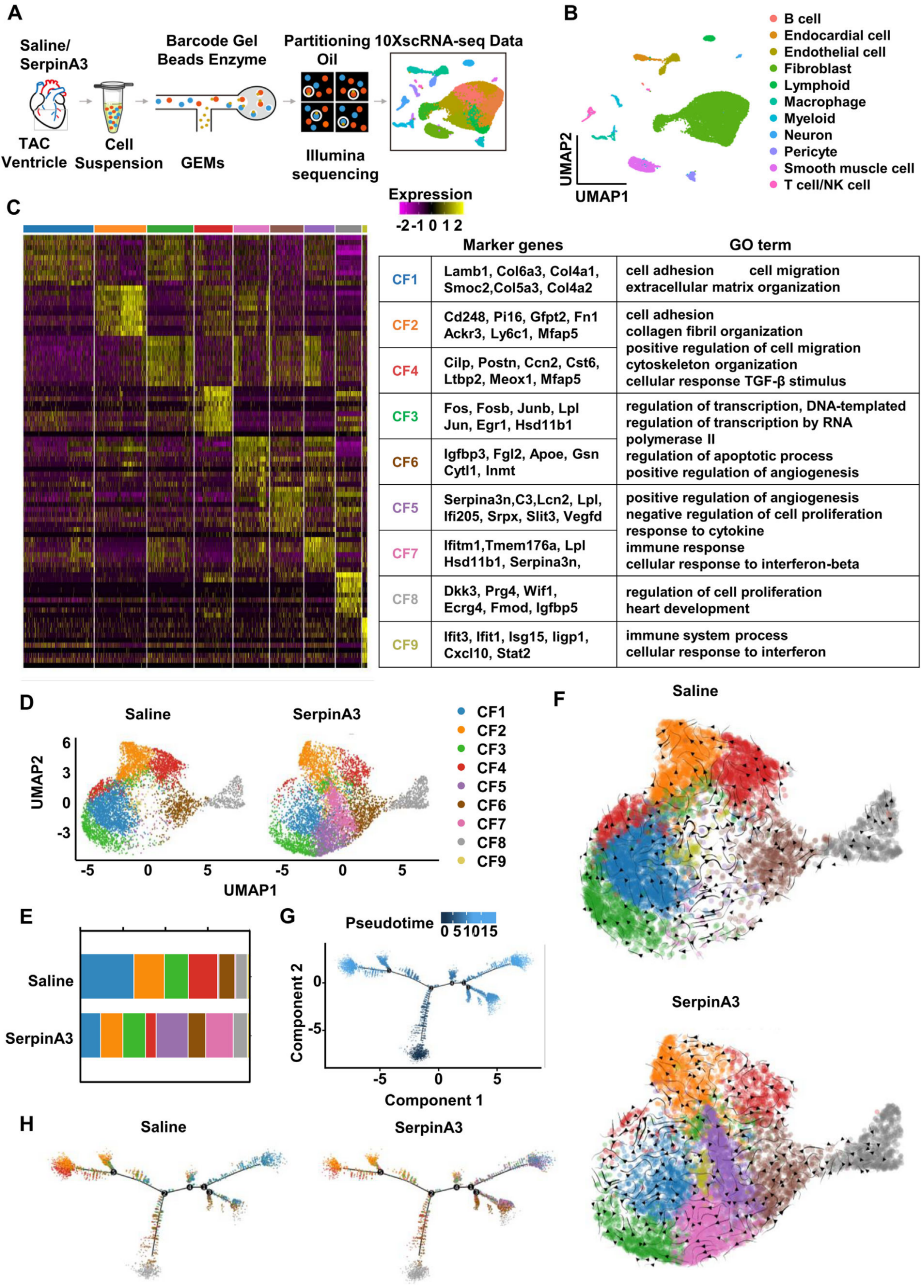
SerpinA3 suppresses cardiac fibroblast activation and myofibroblast differentiation. (A) Schematic of the experimental procedure for isolation and analysis of the left ventricular tissues of mice by single‐cell RNA sequencing (scRNA‐seq). Ventricular tissues were isolated from mice that underwent TAC surgery and were subsequently treated with recombinant human SerpinA3 protein (100ug/kg), as well as from mice that were treated with sterile saline. (B) Uniform manifold approximation and projection (UMAP) integrating 18 388 single cells from Saline and SerpinA3 ventricles. 22 clusters indicated by colors are marked with the presumed cell types. (C) UMAP embedding 13 743 cardiac fibroblasts (CFs) from Saline and SerpinA3 ventricles. Heatmap showing the top 10 markers per cluster in the scRNA‐seq data. 9 clusters (CF1 through CF9) are identified. Representative Gene Ontology (GO) terms and the top marker genes in each CF cluster. (D)UMAP of CFs colored by clusters in the Saline and SerpinA3 groups. (E) Bar plot of the percentage of CFs cluster contributions in each scRNA‐seq sample. (F) Projection of RNA velocities in the Saline and SerpinA3 groups. CF populations onto UMAP plots. The lines with arrows indicate main changes in velocity and estimated future state. (G) Monocle‐generated developmental trajectory. (H) The integration of cell identities within pseudotime from Saline and SerpinA3 groups.

To define the cellular and molecular basis of SerpinA3‐mediated cardiac protection, we performed single‐cell RNA sequencing (scRNA‐seq) of cardiac interstitial cells, with a focus on CFs, using the 10x Genomics platform. Raw sequencing data was processed and aligned to the reference genome; the mapping rate was 95.5% for the  SerpinA3‐treated TAC group and 96.3% for the vehicle‐treated TAC group, indicating high‐quality and reliable sequencing data for subsequent analysis. After quality control filtering and removal of doublets, we obtained transcriptomic profiles from 18 388 single cells with an average of ≈1927 genes per cell derived from vehicle‐treated TAC mice (*n* = 3 ventricles) and SerpinA3‐treated TAC mice (*n* = 3 ventricles; Figure 3A and Figure S2G and Table S2).

PCA showed the overall cell spectrum was similar between the two groups, with partial differences in gene expression profiles (Figure S2H). Unsupervised clustering identified 11 major cardiac interstitial and immune cell populations, including fibroblasts (Dcn, Col1a1, Col5a1, and Gas1), pericytes (Kcnj8, Abcc9, Vtn, and Colec11), smooth muscle cells (Cnn1, Myh11, Tagln, and Pln), endocardial cells (Npr3, Ptgs1, Cgnl1, and H19), endothelial cells (Ptprb, Cdh5, Fabp4, and Ly6c1), B cells (Ly6d, Cd79b, Ms4a1, and Iglc3), lymphatic endothelial cells (Mmrn1, Lyve1, Flt4, and Ccl21a), macrophages (Pld4, Csf1r, Ms4a6c, and C1qa), myeloid cells (Slpi, Il1b, S100a9, and Ngp), neurons (Kcna1, Nrn1, Plp1, and Lgi4), and T/NK cells (Skap1, Trbc2, Nkg7, and Ms4a4b) (Figure 3B and Figure S3A,B). The overall proportion of CFs was modestly reduced following SerpinA3 treatment compared with vehicle (74.31% vs. 75.26%; Figure S3C).

We next interrogated the CF compartment to delineate the antifibrotic mechanism of SerpinA3. Sub‐clustering analysis resolved nine distinct CF subsets (Figure 3C). CF3, CF5, CF6, and CF7 expressed markers of quiescent or steady‐state fibroblasts, including Hsd11b1, Lpl, Smoc2, Slit3, and Inmt [14, 15]. The CF1 population was enriched in genes associated with extracellular matrix organization, whereas CF2 and CF4 expressed genes related to activated fibroblasts; CF2 highly expressed genes including Mfap5, Pi16, and Fn1, CF4 highly expressed genes including Cilp, Postn, Ccn2, and Meox1 [16]. CF8 expressed Wif1, consistent with an endocardial‐derived fibroblast identity [17, 18]. Whereas CF9 was enriched for interferon‐responsive genes (Ifit3, Ifi203, and Iigp1), indicative of an inflammatory fibroblast phenotype [19] (Figure 3C). Quantitative analysis revealed a marked reduction in activated and myofibroblast subsets in SerpinA3‐treated mice compared with vehicle‐treated TAC controls (CF1: 11.90% vs. 31.57%; CF2: 13.16% vs. 17.98%; CF4: 6.26% vs. 17.27%), accompanied by a substantial expansion of quiescent/steady‐state fibroblasts (58.93% vs. 24.87%) (Figure 3D,E).

To further characterize fibroblast state dynamics, we performed RNA velocity analysis. In saline‐treated TAC hearts, CF trajectories were predominantly confined to activated (CF2 and CF4) and ECM–producing states (CF1). In contrast, SerpinA3 treatment preserved fibroblasts in a quiescent state, and promoted transitions toward distinct CF subsets (CF5 and CF7) (Figure 3F). Consistent with these findings, pseudotime trajectory analysis delineated the progression of CF differentiation (Figure 3G). Quiescent fibroblast subsets (CF3, CF6, and CF8) were positioned at the origin of the trajectory and transitioned toward activated fibroblast populations (CF2 and CF4) and ECM‐enriched fibroblasts (CF1) in TAC hearts. Notably, SerpinA3 treatment markedly reduced these transitions toward activated states and instead favored differentiation toward quiescent fibroblast subsets, including CF5 and CF7, which are associated with pro‐angiogenic features (Figure 3H and Figure S3D).

### 2.4 SerpinA3 Suppresses TGF‐β Signaling Pathway and TGF‐β Induced‐Cardiac Fibroblast Activation

**FIGURE 4:**
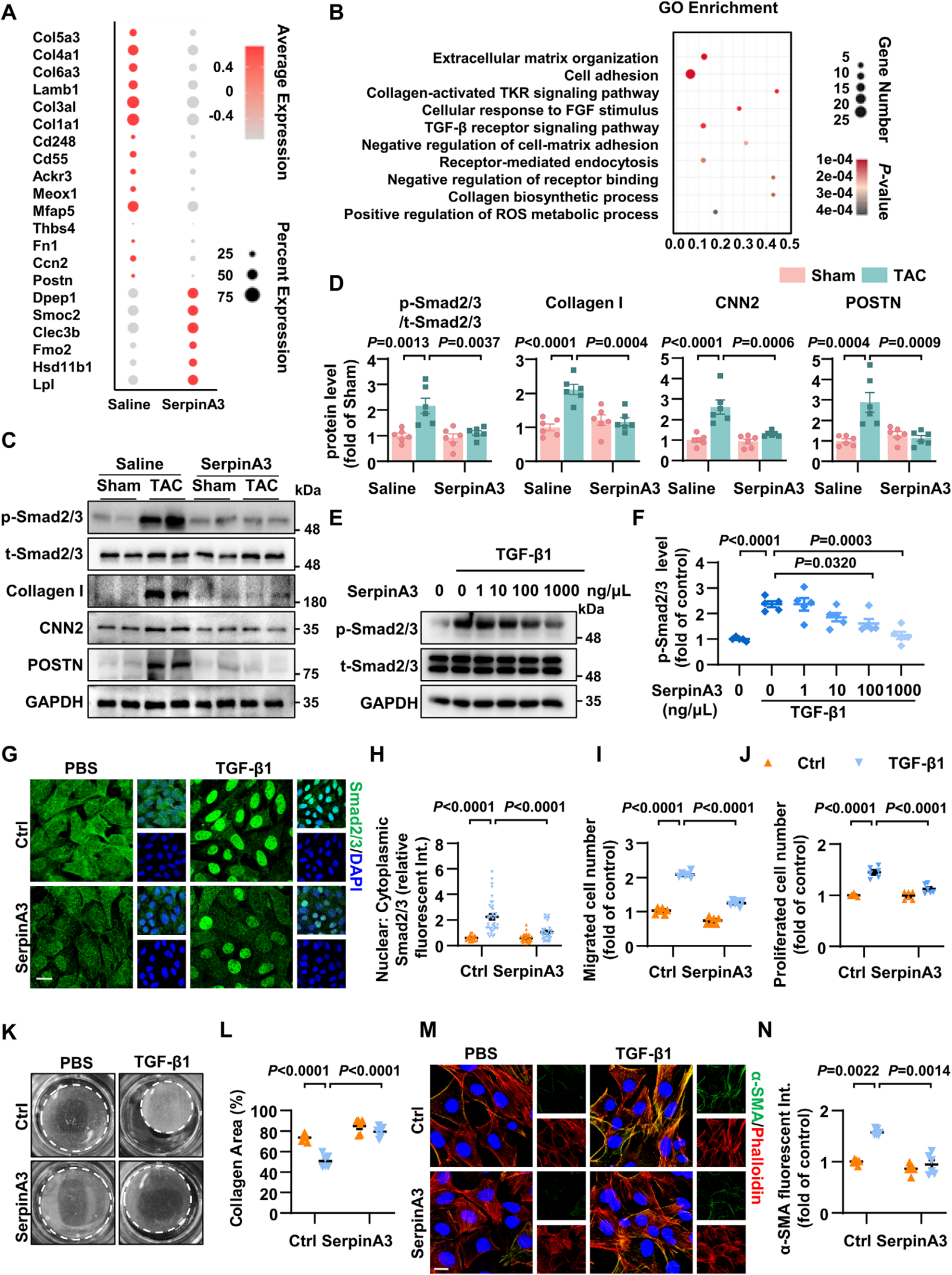
SerpinA3 inhibits cardiac fibroblast activation by blocking TGF‐β signaling. (A) The signature genes for each subcluster are represented by dot plots. The color and size of the dots indicate the relative average expression level in each population and the percentage of cells expressing the gene, respectively. (B) Dot plot representation of GO terms that are derived from differentially expressed genes in the Saline and SerpinA3 groups. (C) C57BL/6J mice underwent Sham or TAC surgery, followed by intraperitoneal injection of recombinant human SerpinA3 protein (100ug/kg) every 3 days for 8 weeks. Protein levels of p‐Smad2/3, t‐Smad2/3, Collagen I, POSTN, and CCN2 in the left ventricle of mice were detected by western blot analysis. (D) Quantification of p‐Smad2/3/t‐Smad2/3, Collagen I, POSTN, and CCN2 in (C). (E) Mouse primary CFs (mPCFs) were pretreated with different concentrations of recombinant human SerpinA3 protein for 30 min, followed by 2 h of TGFβ1 (10 ng/mL) stimulation. Protein levels of p‐Smad2/3 and t‐Smad2/3 were detected by western blot analysis. (F) Quantification of p‐Smad2/3/t‐Smad2/3 in (E) (*n* = 5 independent experiments). (G) mPCFs were treated with recombinant human SerpinA3 protein (100ug/mL) for 30 min before treatment with TGFβ1 (10 ng/mL) for 2 h. Immunofluorescence staining of total‐Smad2/3 (green) and DAPI (blue). Scale bars: 20 µm. (H) The ratio of the nuclear/ cytoplasmic fluorescence intensities of total‐SMAD2/3 in (G). (I) mPCFs migration was assessed by Transwell migration assay. (J) mPCFs proliferation was assessed by CCK‐8 assay. (K) The contractility of mPCFs was assessed by Gel contraction assay. (L) Quantification of the shrinkage area of the gel in (K). (M) Representative confocal microscopy images of immunofluorescence stained for a‐SMA (green), phalloidin (red), and DAPI (blue). Scale bars: 20 µm. (N) Quantification of α‐SMA fluorescence intensity in (M). Data are mean ± SEM, *n* = 6 independent experiments, 2‐way ANOVA with Bonferroni post‐test.

To define transcriptional alterations underlying the antifibrotic effects of SerpinA3, we performed differential gene expression analysis of cardiac fibroblasts (CFs) from SerpinA3‐treated TAC mice and vehicle‐treated TAC controls. Notably, genes encoding extracellular matrix components (*Col1a1*, *Col3a1*, and *Fn1*) and profibrotic mediators (*Postn*, *Ccn2*, and *Mfap5*) were significantly downregulated, whereas markers associated with quiescent fibroblasts (*Dpt*, *Hsd11b1*, and *Lpl*) were upregulated in SerpinA3‐treated CFs (Figure 4A). Consistent with the single‐cell findings, quantitative PCR analysis confirmed that TAC‐induced upregulation of *Col1a1*, *Postn*, and *Ccn2* in whole cardiac tissue was markedly attenuated by SerpinA3 supplementation (Figure S4A).

Gene Ontology enrichment analysis of genes downregulated by SerpinA3 revealed significant association with fibroblast activation–related pathways, including Extracellular matrix organization, Cell adhesion, TGF‐β receptor signaling pathway, Collagen biosynthetic process and et al. (Figure 4B). Among these pathways, TGF‐β receptor signaling pathway emerged as a prominently enriched term, suggesting that SerpinA3 modulates fibroblast activation through suppression of canonical TGF‐β signaling.

Consistent with this notion, western blotting analyses demonstrated that SerpinA3 treatment significantly reduced Smad2/3 phosphorylation in the hearts of TAC mice, accompanied by decreased expression of extracellular matrix proteins including CCN2, POSTN, and collagen I (Figure 4C,D and Figure S4B). To directly assess the impact of SerpinA3 on TGF‐β signaling in fibroblasts, primary mouse cardiac fibroblasts (mPCFs) were treated with recombinant human SerpinA3 at increasing doses in the presence of TGF‐β1. SerpinA3 suppressed TGF‐β1–induced Smad2/3 phosphorylation in a dose‐dependent manner (Figure 4E,F and Figure S4C) and markedly inhibited nuclear translocation of Smad2/3 (Figure 4G,H).

We next evaluated the functional consequences of SerpinA3 on fibroblast activation. TGF‐β1 robustly induced expression of canonical fibrotic genes, including Col1a1, Ccn2, and Postn, which was significantly blunted by SerpinA3 treatment (Figure S4D). Functional assays further corroborated the inhibitory effects of SerpinA3 on CF activation. Transwell migration assays showed that SerpinA3 impaired mPCF migratory capacity and reversed TGF‐β1–induced migration (Figure 4I and Figure S4E,F), while CCK‐8 assay revealed reduced fibroblast proliferation (Figure 4J). Moreover, SerpinA3 significantly attenuated TGF‐β1–induced collagen gel contraction, indicating suppression of fibroblast contractile activity (Figure 4K,L). Consistently, immunofluorescence staining demonstrated that SerpinA3 inhibited TGF‐β1–induced myofibroblast phenoconversion in mPCFs (Figure 4M,N).

### 2.5 SerpinA3 Binds TGFR‐1 and Disrupts TGFR‐1–TGFR‐2 Complex Formation

**FIGURE 5:**
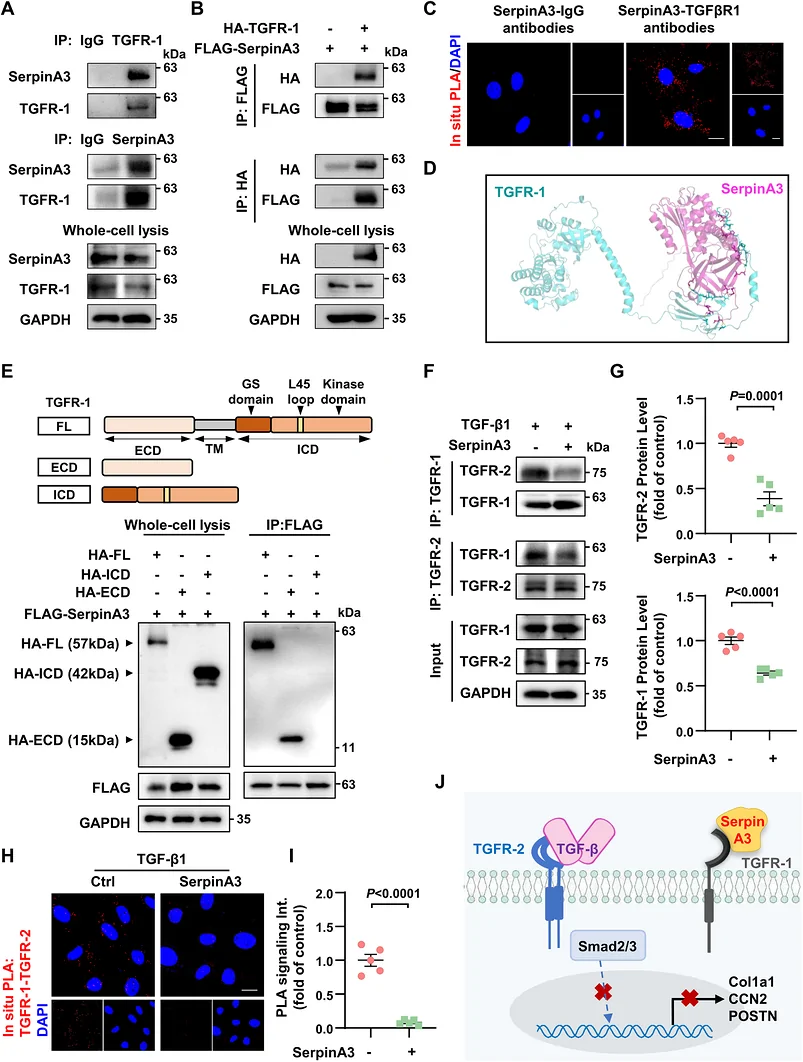
SerpinA3 inactivates TGF‐β signaling by binding the extracellular domain of TGFR‐1 and disrupting receptor complex formation. (A) Whole cell lysates of mPCFs were immunoprecipitated and then immunoblotted with antibodies against the indicated proteins, IgG was used as a negative control. (B) HEK293T cells were co‐transfected with the FLAG‐SerpinA3 plasmid and HA‐TGFR‐1 plasmid for 48 h. Whole cell lysates were immunoprecipitated with anti‐Flag or anti‐HA immunomagnetic beads and immunoblotted with the indicated antibodies. (C) Proximity ligation assay (PLA) assay of SerpinA3 and TGFR‐1 on mPCFs using specific antibodies; IgG was used as a negative control. (D) Molecular docking of SerpinA3 and TGFR‐1 was performed by Cluspro2.0 and PyMol (Python Molecular Viewer; version 2.6.0) was used to display the binding location between SerpinA3 and TGFR‐1. (E) Domain architecture of the TGFR‐1 protein full length (FL), TGFR‐1 extracellular domain (ECD), and TGFR‐1 intracellular domain (ICD). HEK293T cells were co‐transfected with the Flag‐SerpinA3 plasmid and HA‐FL, HA‐ECD, and HA‐ICD plasmids for 48 h. Whole cell lysates were immunoprecipitated with anti‐Flag immunomagnetic beads and immunoblotted with the indicated antibodies. (F) mPCFs were treated with recombinant human SerpinA3 protein (100ug/mL) for 30 min before treatment with TGFβ1 (10 ng/mL) for 2 h. Whole cell lysates were immunoprecipitated with TGFR‐1 or TGFR‐2 antibody and immunoblotted with TGFR‐2 and TGFR‐1 antibody. (G) Quantification of TGFR‐1‐bound TGFR‐2 and TGFR‐2‐bound TGFR‐1 in (F). (H) The levels of the TGFR‐1‐TGFR‐2 protein complex were determined by PLA. (I) Quantification of fluorescence intensity in (H). Data are mean ± SEM, *n* = 5 independent experiments, unpaired 2‐tail *t* test. (J) Schematic diagram depicting the underlying mechanism.

To elucidate the mechanism by which SerpinA3 inhibits TGF‐β signaling, we investigated whether SerpinA3, as a secreted protein, directly interacts with TGF‐β receptors. Co‐immunoprecipitation (co‐IP) assays in mPCFs demonstrated a specific interaction between endogenous SerpinA3 and TGFR‐1 (Figure 5A). This interaction was independently validated in HEK293T cells co‐transfected with HA‐tagged TGFR‐1 and FLAG‐tagged SerpinA3, in which immunoprecipitation using either HA or FLAG antibodies consistently detected SerpinA3–TGFR‐1 binding (Figure 5B).

To further confirm this interaction in situ, we performed a fluorescence‐based proximity ligation assay (PLA), which revealed a robust SerpinA3–TGFR‐1 interaction at the cellular level (Figure 5C). Structural modeling of the SerpinA3–TGFR‐1 complex using Cluspro2.0, and PyMol predicted a stable interaction between SerpinA3 (AlphaFold DB: AF‐P01011‐F1, purple) and TGFR‐1 (AlphaFold DB: AF‐P36897‐F1, green) (Figure 5D). To define the binding interface, we generated full‐length and truncated TGFR‐1 constructs encompassing either the extracellular domain (TGFR‐1‐ECD) or intracellular domain (TGFR‐1‐ICD). Co‐IP assays demonstrated that SerpinA3 selectively interacted with TGFR‐1‐ECD, but not with TGFR‐1‐ICD, indicating that SerpinA3 binds the extracellular region of TGFR‐1 (Figure 5E).

Given that canonical TGF‐β signaling requires ligand‐induced assembly of a heterotetrameric complex composed of TGFR‐1 and TGFR‐2, we next examined whether SerpinA3 affects receptor complex formation. In mPCFs stimulated with TGF‐β1, co‐IP assays revealed that SerpinA3 markedly reduced TGF‐β–induced recruitment of TGFR‐1 to TGFR‐2 (Figure 5F,G). Consistently, PLA analysis demonstrated a significant decrease in TGFR‐1–TGFR‐2 proximity in the presence of SerpinA3 under TGF‐β stimulation (Figure 5H,I).

Collectively, these findings indicate that SerpinA3 binds the extracellular domain of TGFR‐1, thereby impairing TGFR‐1–TGFR‐2 complex formation and attenuating downstream TGF‐β signaling in cardiac fibroblasts (Figure 5J).

### 2.6 Cardiac SerpinA3 Overexpression Attenuates Pressure Overload–Induced Cardiac Dysfunction and Remodeling

**FIGURE 6:**
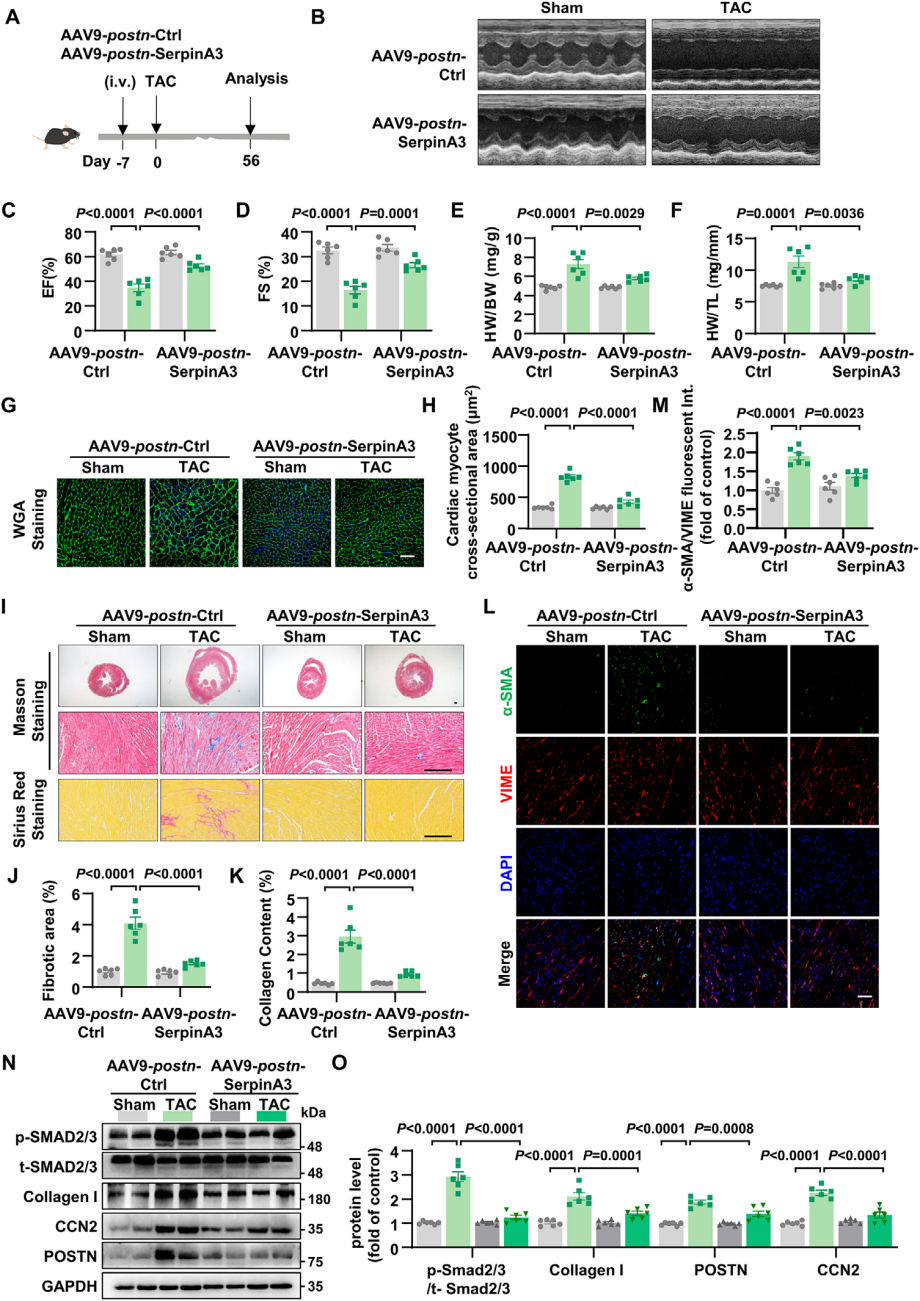
Myofibroblast‐targeted AAV‐mediated SerpinA3 overexpression ameliorates pressure overload–induced cardiac remodeling. (A) C57BL/6J mice were injected with AAV9‐*postn*‐Ctrl and AAV9‐*postn*‐SerpinA3 via the tail vein and underwent Sham or TAC surgery for 8 weeks. (B) M‐mode echocardiographic images of 4 groups mice. (C, D) Echocardiographic analysis of left ventricular ejection fraction (EF) and fractional shortening (FS) in each group. (E, F) Ratio of heart weight (HW) to body weight (BW), and ratio of heart weight (HW) to tibial length (TL) in each group. (G) Representative images of Gated wheat germ agglutinin (WGA) staining of the left ventricle. Scale bar: 50 µm. (H) Quantification of the cross‐sectional area of the left ventricular cardiomyocytes in (G). (I) Representative images of Masson staining and Sirius red staining of the left ventricle. Scale bar: 200 µm. (J, K) Quantification of fibrotic area and collagen content of the left ventricular in (I). (L) Representative confocal microscopy images of immunofluorescence staining for a‐SMA (green) and vimentin (VIME, fibroblast marker) (red). Scale bar: 50 µm. (M) Quantification of co‐localization of a‐SMA and VIME fluorescence intensity in (L). (N) Protein levels of p‐SMAD2/3, t‐SMAD2/3, Collagen I, POSTN, and CCN2 in the left ventricle of mice detected by western blot analysis. (O) Quantification of p‐SMAD2/3/t‐Smad2/3, Collagen I, POSTN, and CCN2 in (N). Data are mean ± SEM, n = 6 mice per group, 2‐way ANOVA with Bonferroni post‐test.

Given the therapeutic potential of gene‐based strategies, we next evaluated whether targeted cardiac overexpression of SerpinA3 could mitigate pressure overload–induced cardiac remodeling. To selectively target myofibroblasts and minimize potential adverse effects associated with excessive inhibition of TGF‐β signaling in other cardiac cell types, we generated an adeno‐associated virus serotype 9 (AAV9) vector encoding full‐length human SerpinA3 under the control of the myofibroblast‐specific POSTN promoter (AAV9‐*Postn*‐SerpinA3). An empty AAV9 vector served as control (AAV9‐*Postn*‐Ctrl). Mice were systemically injected with AAV9‐Postn‐SerpinA3 or the control vector and subsequently subjected to TAC for 8 weeks (Figure 6A).

After 8 weeks, SerpinA3 overexpression significantly improved cardiac function, as evidenced by restoration of EF and FS (Figure 6B–D), and attenuated TAC‐induced increases in IVSTd, LVPWd, LVIDs, LVIDd and LV mass (Figure S5A–E). Consistently, HW/BW and HW/TL ratios were significantly reduced in SerpinA3‐overexpressing TAC mice compared with controls (Figure 6E,F). Histological analyses further demonstrated that SerpinA3 overexpression attenuated cardiac hypertrophy, as shown by WGA staining (Figure 6G,H), and markedly reduced myocardial fibrosis and collagen deposition, as assessed by Masson's trichrome and Sirius Red staining (Figure 6I–K). Immunofluorescence analysis revealed that SerpinA3 overexpression suppressed TAC‐induced α‐SMA expression in vimentin‐positive cardiac fibroblasts, indicating reduced fibroblast activation (Figure 6L,M). Consistent with inhibition of TGF‐β signaling, immunoblot analysis showed that SerpinA3 overexpression significantly reduced Smad2/3 phosphorylation in TAC hearts, accompanied by decreased mRNA and protein expression of extracellular matrix proteins, including CCN2, POSTN, and collagen I (Figure 6N,O and Figure S5F,G). Collectively, these findings demonstrate that AAV9‐mediated SerpinA3 overexpression preserves cardiac function and attenuates pathological remodeling in response to pressure overload.

## 3 Discussion

In this study, we identify SerpinA3 as a previously unrecognized regulator of pressure overload–induced cardiac remodeling and heart failure. Pharmacological supplementation or genetic augmentation of SerpinA3 markedly attenuated cardiac dysfunction and fibrotic remodeling in response to pressure overload. Single‐cell transcriptomic analyses further demonstrated that SerpinA3 suppresses profibrotic fibroblast programs by promoting a transition from activated and extracellular matrix–producing fibroblasts toward a quiescent, pro‐angiogenesis state. Mechanistically, our data indicate that TGF‐β signaling contributed to the protective role of SerpinA3. We show that SerpinA3 functions as an endogenous antagonist of TGFR‐1, binding its extracellular domain and disrupting TGFR‐1–TGFR‐2 complex formation, thereby preventing downstream Smad2/3 phosphorylation in cardiac fibroblasts. This receptor‐level interference provides a mechanistic basis for the broad antifibrotic effects of SerpinA3 observed in vivo and at single‐cell resolution. Collectively, these findings establish SerpinA3 as a critical modulator of fibroblast activation and fibrotic remodeling in pressure overload–induced heart failure, and suggest that targeting SerpinA3–TGFR‐1 interactions may represent a therapeutic strategy for limiting pathological cardiac fibrosis.

SerpinA3, also termed α‐1‐antichymotrypsin (AACT), is an acute‐phase protease inhibitor predominantly synthesized by hepatocytes and released into the circulation [20, 21]. Beyond its canonical hepatic functions, SerpinA3 has been implicated in the regulation of cellular proliferation and differentiation [22]. Changes in SerpinA3 protein levels in chronic and acute heart disease have been well documented in recent years [23]. Circulating SerpinA3 levels rise transiently after acute myocardial infarction, reflecting its systemic acute‐phase response. In humans, the single SerpinA3 gene is represented in mice by a cluster of 14 paralogs [24]. Among these, SerpinA3K was originally identified for its specific inhibitory effect on tissue kallikrein and is expressed in the liver, kidney, and ocular tissues. Beyond classical protease inhibition, SerpinA3K exhibits anti‐neovascular, anti‐inflammatory, and antioxidative effects in retinal tissue [25, 26]. In our study, SerpinA3K emerged as one of the most downregulated proteins in TAC‐induced failing hearts. These findings are consistent with human left ventricular RNA‐seq and proteomic data, which show decreased SerpinA3 expression in failing hearts [22, 27, 28, 29]. Recent evidence further supports a cardioprotective role for SerpinA3K in limiting cardiomyocyte apoptosis following ischemia–reperfusion injury [30]. Given that cardiomyocyte hypertrophy in TAC models secondarily promotes fibroblast activation and interstitial fibrosis, cardiomyocytes may also contribute indirectly to the overall protective effects of SerpinA3. Other murine SerpinA3 paralogs, including SerpinA3C and SerpinA3N, similarly exhibit cardioprotective effects, and their deletion exacerbates cardiac fibrosis [31, 32]. Mechanistically, SerpinA3C translocated into the nucleus of cardiac fibroblasts and interacts with the nuclear receptor Nr4a1, promoting its acetylation and thereby suppressing Enolase 1 transcription and pathological glycolytic activation, ultimately restraining fibroblast proliferation and myofibroblast differentiation after myocardial infarction. Consistently, conditional deletion of SerpinA3N results in increased fibrosis and inflammation, accompanied by impaired contractile function in infarcted hearts. In line with these observations, administration of recombinant human SerpinA3 in our TAC model significantly attenuated pressure overload–induced cardiac dysfunction, hypertrophy, and fibrosis. Together, these findings extend prior human and murine studies by establishing a direct functional role for SerpinA3 in modulating pathological cardiac remodeling in vivo.

Cardiac fibrosis can be divided into two categories, reparative fibrosis and reactive fibrosis, each of which can be recapitulated by several models of heart failure. The TAC‐induced left ventricular pressure overload animal model is characterized initially by reactive interstitial fibrosis, an adaptive response that preserves cardiac structure and function, followed by replacement fibrosis in areas of cardiomyocyte necrosis [33]. Sustained activation of cardiac fibroblasts and excessive extracellular matrix deposition are central drivers of myocardial stiffening and increased ventricular wall stress [34]. Elevated wall stress, in turn, serves as a potent biomechanical stimulus that promotes the transition from adaptive to maladaptive cardiac hypertrophy, ultimately accelerating heart failure progression. Indeed, accumulating evidence from hypertrophic cardiomyopathy and other forms of cardiac remodeling suggests that fibrosis is not merely a secondary consequence of disease but an active contributor to pathological remodeling, with profibrotic gene programs and collagen synthesis becoming activated before overt left ventricular hypertrophy develops [35, 36]. In the present study, SerpinA3 attenuated both cardiac fibroblast activation and cardiomyocyte hypertrophy in response to pressure overload. Moreover, treatment with recombinant SerpinA3 improved survival compared with saline‐treated TAC mice. Given the central role of fibrosis in determining myocardial stiffness and ventricular wall stress, it is plausible that suppression of fibroblast activation and extracellular matrix accumulation represents a major mechanism underlying the cardioprotective effects of SerpinA3. By limiting pathological fibrosis, SerpinA3 may reduce the mechanical burden imposed on the myocardium, thereby attenuating maladaptive hypertrophic remodeling and preserving cardiac function. Moreover, recent studies have shown that lactylated SerpinA3K secreted from CFs protects cardiomyocytes from I/R‐induced apoptosis via WNT pathway inhibition [30]. As multiple lines of evidence link both canonical and non‐canonical WNT pathways to maladaptive cardiac hypertrophy [37], a direct anti‐hypertrophic effect of SerpinA3K on cardiomyocytes cannot be excluded.

The TGF‐β family of growth factors is perhaps the most extensively studied mediator of fibroblast activation, with TGF‐β1 likely playing the greatest role in pathological fibrosis [9, 33]. TGF‐β1 signals through stepwise assembly of heterotetrameric receptor complexes composed of type II and type I serine/threonine kinases. Upon ligand binding, TGF‐β1 initially engages the high‐affinity TGFR‐2, which subsequently recruits the low‐affinity TGFR‐1 (also known as activin receptor–like kinase 5, ALK5) [11]. The constitutively active TGFR‐2 then phosphorylates TGFR‐1 within its juxtamembrane GS domain, inducing conformational changes that promote displacement of the inhibitory protein PPIase FKBP1A and enable docking and phosphorylation of receptor‐activated SMAD2 and SMAD3 [38]. Activated SMADs translocate to the nucleus to drive transcriptional programs governing fibroblast activation and extracellular matrix production [9, 11].

Pharmacological inhibition of the TGFR‐1 has been extensively explored as an antifibrotic strategy [33]. The orally active TGFR‐1 inhibitor GW788388 attenuated left ventricular remodeling and improved cardiac function in a rat model of myocardial infarction [39]. Similarly, SM16 abolished SMAD2 phosphorylation induced by aortic banding in vivo and by TGF‐β in cultured cardiac fibroblasts, leading to reduced extracellular matrix synthesis, diminished collagen cross‐linking, and decreased myocardial stiffness. These effects were associated with improved diastolic function and cardiac output, accompanied by reductions in lung congestion and atrial natriuretic peptide expression. However, despite these favorable functional outcomes, SM16 treatment was associated with increased mortality, progressive left ventricular dilatation, and inflammatory valvular lesions, raising significant safety concerns that may limit the translational potential of small‐molecule TGFR‐1 inhibitors [40]. Consistently, pan–TGF‐β neutralizing antibodies have been reported to induce cardiovascular toxicity in preclinical models [41], and the Phase 2 clinical trials of the TGFR‐1 inhibitor galunisertib excluded patients with moderate to severe cardiac disease [42, 43]. These limitations have motivated alternative strategies aimed at modulating TGF‐β signaling at the receptor–ligand interface rather than through systemic kinase inhibition [11]. In this study, our findings identify SerpinA3 as an endogenous regulator of TGF‐β signaling in the heart. By binding the extracellular domain of TGFR‐1, SerpinA3 disrupts TGFR‐1–TGFR‐2 complex assembly, thereby preventing downstream SMAD2/3 activation in cardiac fibroblasts. Unlike small‐molecule kinase inhibitors, SerpinA3 acts locally within the cardiac interstitium and is secreted by fibroblasts themselves, providing a physiologically tuned and spatially constrained blockade of profibrotic signaling. This receptor‐level modulation selectively suppresses fibroblast activation while preserving essential systemic TGF‐β functions, supporting SerpinA3 as a potentially safer therapeutic strategy to limit pathological fibrosis in pressure overload–induced heart failure.

In conclusion, our study identifies SerpinA3 as a previously unrecognized endogenous brake on profibrotic TGF‐β signaling in the heart. By directly binding the extracellular domain of TGFR‐1 and preventing TGFR‐1–TGFR‐2 complex formation, SerpinA3 suppresses SMAD2/3 activation and fibroblast phenotypic modulation, thereby limiting pathological extracellular matrix deposition and adverse cardiac remodeling under pressure overload.

## 4 Materials and Methods

### 4.1 Animals

All study protocols for animal experiments were approved by the Institutional Animal Care and Use Committee of Tianjin Medical University and conformed to the current NIH guidelines for Care and Use of Laboratory Mice were housed under controlled conditions (20°C ± 2°C; 12 h light/dark cycle) with ad libitum access to food and water. TAC surgery was performed as previously described [12]. Male wild‐type C57BL/6J mice aged 9–10 weeks were randomly assigned to sham surgery and TAC surgery. Prior to surgery, a small animal ventilator was connected, and a local thoracotomy was performed at the second intercostal space on the left chest. The left common carotid artery, unnamed artery (right brachiocephalic artery), and aortic arch were exposed under a stereoscope, and the constriction location was selected at the aortic arch between the left common carotid artery and unnamed artery. A 27G needle was used as a spacer, and then the aortic arch was ligated with 6/0 monofilament suture. The sham operation group underwent the same surgical procedure but without ligation. After the operation, the mice were placed in pre‐warmed cages for recovery and postoperative monitoring. After awakening, recombinant SerpinA3 protein (100ug/kg) (RP00194; ABclonal, Wuhan, CN) was intraperitoneally injected every 3 days for 8 weeks. The animal numbers for each experiment are provided in the figure legends.

### 4.2 TMT (tandem mass tag) Proteomics

The proteomic experiment was performed by Shanghai Genechem Co., Ltd. Proteins were extracted using the homogenization‐SDT lysis method, and protein concentration was determined by the bicinchoninic acid (BCA) assay. According to the manufacturer's instructions, peptide mixtures were labeled with TMT 10plex Isobaric Mass Tag Labeling Kit (Thermo Fisher Scientific), achieving 99.2% labeling efficiency. Chromatographic separation was performed on an Easy‐nLC 1200 nanoflow system (Thermo Fisher Scientific). The analytical column (Acclaim PepMap RSLC C18, 50 µm × 15 cm, nano Viper, P/N164943) was equilibrated with 100% mobile phase A (0.1% formic acid). Samples were loaded via autosampler and eluted with a linear gradient of mobile phase B (80% acetonitrile and 0.1% formic acid) at a flow rate of 300 nL/min. LC‐MS/MS analysis was conducted on a Q‐Exactive Plus hybrid quadrupole‐Orbitrap mass spectrometer (Thermo Fisher Scientific) operated in positive ion mode. MS/MS raw files were processed using the MASCOT 2.6 search engine (Matrix Science) embedded in Proteome Discoverer 2.1, and searched against the UniProt database. The search parameters included trypsin as the enzyme used to generate peptides with a maximum of two missed cleavages permitted. A precursor mass tolerance of 10 ppm was specified and 0.05 Da tolerance for MS2 fragments. A peptide false discovery rate of 1% was enforced using a decoy database strategy. PCA was performed to evaluate the clustering trends and repeatability among samples based on the normalized protein expression matrix. Proteins with Fold change>1.2 and *p*‐value (Student's *t*‐test) <0.05 were differentially expressed proteins. The proteomics results are shown in Table S3. The mass spectrometry proteomics data have been deposited to the ProteomeXchange Consortium (https://proteomecentral.proteomexchange.org) via the iProX partner repository [44, 45] with the dataset identifier PXD079845.

### 4.3 Single Cell Sequencing

We prepared male mice from TAC‐treated mice with sterile saline and recombinant SerpinA3 protein respectively for single‐cell sequencing. Single‐cell suspensions from isolated mouse left ventricles were prepared by tissue mincing and enzyme digestion as previously described. Briefly, mice were euthanized and subsequently perfused with sterile saline. The left ventricles were isolated and digested. The obtained cell suspensions were filtered twice through 70 µm cell strainers and then resuspended in phosphate‐buffered saline (PBS) containing 0.04% BSA. Finally, Cell suspensions were loaded into the 10×Genomics Chromium instrument for library preparation.

The scRNA‐seq data were generated by LC‐Bio Technology Co., Ltd (Hangzhou, China) and Bioinformatic analysis was performed using the OmicStudio tools at https://www.omicstudio.cn/tool. Raw sequencing data were processed using Cell Ranger (9.0.1) software and aligned to the reference genomes. The Seurat (version 4.5.2) R package was applied to perform quality control (QC) and analysis of the single‐cell data. To obtain high‐quality scRNA‐seq data, cells were filtered for mitochondrial reads per cell (percent.mito) <25% and 500< detected genes per cell (nFeature_RNA). Then, the single‐cell expression matrix was standardized after QC and screening for highly variable genes. PCA was performed based on the expression matrix of highly variable genes, mapping high‐dimensional expression data to a low‐dimensional space to extract the dominant transcriptional variation among cells. Significant principal components were screened based on the contribution of each principal component to the overall variation, and combined with the results of the Elbow Plot, which were further used for subsequent cell clustering, UMAP dimensionality reduction, and cell subtype identification. A bimod test was employed for cell clustering and marker gene analysis respectively. To determine gene set's enrichment status and significance, pathway analysis (KEGG database) and Gene Ontology analysis identified significant pathways and biological processes of differential genes via a Wilcox test (*p*‐value and FDR as thresholds). Based on the different gene expression profiles of cells in different states, a temporal map of cell differentiation processes was constructed for pseudo‐temporal analysis. The single‐cell RNA‐seq data generated in this study have been deposited in NCBI's Gene Expression Omnibus under accession code GSE335919.

### 4.4 Adeno‐Associated Virus (AAV) Construction and Infection

The AAV9 carrying a periostin promoter used in this study was constructed as previously reported [46]. AAV9 carrying a periostin promoter driving the expression of SerpinA3 (NM_001085.5) was constructed by Shanghai Genechem Co. (Shanghai, China). The mice were injected into the tail vein with AAV9‐Postn‐SerpinA3 or AAV9‐Ctrl after TAC or sham surgery (5e + 10^11^v.g).

### 4.5 Echocardiography

Trans‐thoracic echocardiography was performed on all mice by using a Vevo 2100 system with a MS400 linear array transducer (VisualSonics, ON, Canada) as previously reported. Briefly, mice were anesthetized with 2% isoflurane and kept warm on a heated platform (37°C). The chest hairs were removed by using depilatory cream, and a layer of acoustic coupling gel was applied to the thorax. An average of 10 cardiac cycles of standard 2‐D and M‐mode short axis at mid‐papillary muscle level were analyzed. Left ventricular ejection fraction and dimensions were calculated by using a modified Quinone method.

### 4.6 Histological Analysis and Immunohistochemistry

Tissues were fixed in 4% paraformaldehyde for 24 h at room temperature and embedded in paraffin. Hearts were sectioned at 5 µm for staining. Collagen deposition was stained with Masson's trichrome (Sigma–Aldrich, MO) and Sirius red (Solarbio Life Sciences, Beijing) according to the manufacturer's instructions. Images of sections were captured under an Olympus inverted microscope (IX53, Tokyo) and fibrotic areas were semi‐quantitatively determined by using ImageJ 1.52.

### 4.7 Immunofluorescence Staining

Sections measuring 5 µm were cut from a paraffin‐embedded tissue block. Then, sections were stained with primary antibodies for Vimentin (ab92547; Abcam, Cambridge, UK), α‐SMA (A2547; Thermo Fisher Scientific, Waltham, MA, USA), CD31 (ab24590; Abcam, Cambridge, UK), α‐actinin (ab90421; Abcam, Cambridge, UK) or SerpinA3K (55480‐1‐AP; Proteintech, Wuhan, CN) overnight at 4°C. Alex 594‐conjugated goat anti‐rabbit antibodies (8889S; Cell Signaling Technology, Danvers, MA, USA) and Alex 488‐conjugated goat anti‐mouse antibodies (4408S; Cell Signaling Technology, Danvers, MA, USA) were used as secondary antibodies. Nuclei were stained with DAPI. Images were acquired under an Olympus inverted microscope (IX81, Tokyo). Fluorescent intensity was quantified using ImageJ.

### 4.8 Western Blotting

Cells and tissues were harvested using RIPA lysis buffer (R0010; Solarbio, Beijing, China) containing complete protease inhibitor cocktail and phosSTOP phosphatase inhibitor (Roche, Mannheim, Germany). Extracts containing equal amounts of total protein were separated using SDS‐PAGE and electrophoretically transferred to nitrocellulose membranes (Millipore, Billerica, MA, USA), blocked with TBST containing 5% bovine serum albumin. The membranes were incubated with specific primary antibodies overnight at 4°C with gentle shaking, followed by horseradish peroxidase‐conjugated secondary antibodies for 1 h at room temperature. Analysis involved the primary antibodies including GAPDH (5174T; Cell Signaling Technology, Danvers, MA, USA), P‐Smad2/3 (8828S; Cell Signaling Technology, Danvers, MA, USA), Smad2/3 (8685S; Cell Signaling Technology, Danvers, MA, USA), SerpinA3K (55480‐1‐AP; Proteintech, Wuhan, CN), SerpinA3 (sc‐69983; Santa cruz, Dallas, TX, USA), Collagen I (A1352; ABclonal, Wuhan, CN), POSTN (A14556; ABclonal, Wuhan, CN), CCN2 (25474‐1‐AP; Proteintech, Wuhan, CN), TGFR‐1 (sc‐518018; Santa cruz, Dallas, TX, USA), TGFR‐2 (sc‐17791; Santa cruz, Dallas, TX, USA), FLAG (14793S; Cell Signaling Technology, Danvers, MA, USA), HA (3724S; Cell Signaling Technology, Danvers, MA, USA). Immunoblots were quantified by using ImageJ 1.52.

### 4.9 Total RNA Extraction and Real‐Time PCR Analysis

Total RNA was extracted from cells by using RNA extraction kits (LS1040; Promega, Madison, WI, USA). RNA samples were reverse Transcript with SuperScript and random primers (AT341; Transgene, Beijing, CN). Real‐time PCR involved use of the SYBR Green qPCR Master Mix (AQ602; Transgene, Beijing, CN) and the ABI 7500 Real‐Time PCR System (Life Technologies, Carlsbad, CA, USA). Primer sequences are in Table S4.

### 4.10 Primary Mouse Cardiac Fibroblasts Isolation and Culture

Isolation of mouse primary CFs (mPCFs) was performed as previously reported [46, 47]. Briefly, mouse hearts from freshly euthanized C57BL/6J mice were harvested and minced to 1 mm in cold phosphate‐buffered saline. Minced tissue was subsequently digested with buffer containing collagenase II (#V900892, Sigma–Aldrich, MO) and trypsin (Solarbio Life Sciences, Beijing) at 37°C for 60 min with mild shaking. The supernatants were spun to collect cells. Then cells resuspended in DMEM/F12 (#11320033, Gibco, MA) were plated into dishes and incubated for 2 h. Supernatant was discarded and dishes were replenished with fresh medium. mPCFs were incubated at 37°C in a humidified atmosphere of 5% CO2 and grown to 70–80% confluence. Cells at passages 2 to 3 were used in experiments.

### 4.11 Cell Counting Kit‐8 Assay

mPCFs were treated with recombinant SerpinA3 protein (100ug/mL) for 30 min before treatment with TGF‐β1 (10 ng/mL) for 24 h. Cell counting kit‐8 (CCK‐8) (Dojindo Laboratories, Japan) was used to assess cell proliferation. In each well of the 96‐well plate, 100 µL cell suspension was inoculated. 10 µL of CCK‐8 solution was added to each well, and then the plates were incubated at 37°C for 2 h. The absorbance of each well was measured at 450 nm using a microplate reader.

### 4.12 Gel Contraction Assay

mPCFs were digested with trypsin and centrifuged, then resuspended and counted in DMEM containing 10% fetal bovine serum (FBS), ensuring a final cell concentration of 3 × 10^5 cells/mL for each group. Collagen type I (3 mg/mL) was adjusted with 1.2 µL of 1 m NaOH per 200 µL^2^, then gently mixed with the cell suspension to avoid bubbles. 500 µL of the mixture was transferred to a 24‐well plate and allowed to solidify at room temperature for 20 min. After gelation, 500 µL of either 10% FBS DMEM/F12 supplemented with TGF‐β1 (10 ng/mL) or the same medium containing recombinant SerpinA3 protein (100ug/mL) was added to each well. The 24‐well plate was placed at 37°C in a humidified 5% CO_2_ incubator, and the contraction status of the gel in both groups of cells was observed after 48 h. ImageJ was used for image analysis.

### 4.13 Boyden Chamber Assay

mPCFs were pretreated with recombinant SerpinA3 protein for 30 min, then stimulated with PBS or TGF‐β1 for 24 h respectively. After 24 h, cells were digested and seeded into the upper chamber of a Transwell chamber, and continued to be treated with SerpinA3 and TGF‐β1. Medium containing 10% FBS was placed in the lower chamber as a chemoattractant. After incubation for 6 h at 37°C in a humidified 5% CO_2_ incubator, cells that had migrated to the lower surface of the membrane were fixed with 4% paraformaldehyde for 30 min and stained with DAPI. Nuclei of migrated cells were imaged and counted under a fluorescence microscope.

### 4.14 Wound‐Healing Assay

When the confluence of mPCFs reached more than 80%, a 200 µL sterile pipette tip was used to draw a uniform vertical line across the bottom of the culture plate. Cells were washed three times with PBS to remove cell debris, followed by the addition of medium containing 10% FBS. Subsequently, cells were pretreated with recombinant SerpinA3 protein for 30 min. After pretreatment, cells were stimulated with PBS and TGF‐β1 for 24 h respectively. The scratch width at 0 h was immediately recorded under an inverted microscope at 10× magnification. After 24 h of incubation at 37°C in a humidified 5% CO_2_ incubator, the scratch width was measured again. The scratch length was quantified using ImageJ software.

### 4.15 Plasmid Construction and Transfection

Plasmids encoding the full‐length human TGFR‐1 [FL; aa 1‐503], and 2 domains of TGFR‐1 (i.e., the extracellular domain‐terminal [ECD; aa 1‐126] and the intracellular domain‐terminal [ICD; aa 148‐503]) with an HA tag were constructed for expression in HEK293T cells. A plasmid encoding the full‐length human SerpinA3 [FL; aa 1‐423] with Flag tag was also constructed for expression in HEK293T cells. Then, the Flag‐SerpinA3 plasmid was transfected into HEK293T cells with HA‐ TGFR‐1 FL, HA‐ TGFR‐1 ECD and HA‐ TGFR‐1 ICD respectively by Lipofectamine 3000 (L3000‐015, Thermo Fisher Scientific, Waltham, MA, USA). The transfection procedures followed the manufacturers’ instructions. After 6–8 h, replace the medium with 10% DMEM and continue to incubate for 48 h in a humidified incubator at 37°C and 5% CO_2_.

### 4.16 Immunoprecipitation Assay

Extraction of whole‐cell lysates was described previously. For immunoprecipitation, 1000 µg protein was incubated with 2 µg specific antibodies overnight at 4°C with constant rotation; 40 µL of 50% Protein A/G PLUS‐Agarose beads (Santa Cruz Biotechnology, Dallas, TX) was then added, and the incubation was continued for another 2 h. Beads were then washed five times with the lysis buffer. Between washes, the beads were collected by centrifugation at 2500 rpm for 5 min at 4°C. After a final wash, the supernatant was aspirated and discarded, then the precipitated proteins were eluted from the beads by resuspending in 1 × SDS‐PAGE loading buffer and boiling for 5 min. The resultant materials from immunoprecipitation or cell lysates underwent western blot analysis.

### 4.17 In Situ Proximity Ligation Assay (PLA)

In situ analysis of protein interactions was performed using a Duolink in Situ Red Starter Kit (Cat. No. DUO92101; Sigma–Aldrich, St. Louis, MO, USA) according to the manufacturer's instructions. Briefly, mPCFs were treated with TGF‐β1 with or without recombinant SerpinA3 protein for 2 h. Then, cells were fixed with 4% paraformaldehyde, permeabilized in 0.1% Triton X‐100, and incubated with the primary antibodies overnight at 4°C. After overnight incubation, Duolink PLA Probe (anti‐mouse and anti‐rabbit probes) was applied to the samples for 1 h at 37°C. The next ligation step (30 min, 37°C) allowed the antibody‐conjugated oligonucleotides to form a DNA circle, followed by incubation at 37°C for 100 min with an amplification buffer. After washing, the slides were mounted onto coverslips using Duolink PLA Mounting Medium with DAPI. Images were captured under an Olympus inverted microscope (IX81, Tokyo).

### 4.18 Statistical Analysis

Sample sizes were designed with adequate power according to the literature and our previous studies. Mice were randomized before being assigned to treatment groups. Sample sizes/numbers of replicates are indicated in the figure legends and visualized in the figures (each symbol represents one animal/biological replicate) and data are displayed as mean and SEM. GraphPad Prism (version 9.0) was used for statistical analysis, and only within‐test corrections were made. The data were tested for normality before parametric statistics using the Shapiro‐Wilk normality test (*n*≥6). For normally distributed data, comparisons between two groups were performed using unpaired Student's *t*‐test, and comparisons among three or more groups were performed using one‐way or two‐way ANOVA followed by Bonferroni multiple comparisons test; For non‐normal data or data with a small sample size (*n*<6), comparisons were performed by the Mann‐Whitney U test or the Kruskal‐Wallis test followed by Dunn's multiple comparison test. For data with ≥2 subcategories and a small sample size (*n*<6) where the normality test could not be conclusive, two‐way ANOVA followed by Bonferroni multiple comparisons test was performed. The criterion for statistical significance was *p* < 0.05.

## Author Contributions

**Chunlu Huang**: investigation, validation. **Hui Wang**: investigation, software, data curation, writing – original draft, methodology. **Wanyi Guo**: visualization. **Jinlong He**: funding acquisition, project administration, writing – review and editing, conceptualization. **Jieyu Cui**: investigation, methodology, formal analysis, writing – original draft. **Yi Zhu**: supervision, writing – review and editing. **Kangyin Chen**: writing – review and editing, conceptualization, funding acquisition. **Shuai Shao**: funding acquisition, project administration, resources. **Tong Liu**: supervision.

## Funding

This work was supported by the National Natural Science Foundation of China (82200428, 82370258, 82422006, 82200476, 82570543, 82200322, 32471166, 82270516, 82170482), Natural Science Foundation of Tianjin (24JCYBJC01140, 24JCJQJC00060, 24JCQNJC01240), Tianjin Health Research Project (TJWJ2024MS007, TJWJ2022QN020), Scientific Research Program of Tianjin Municipal Education Commission (2025ZD007, 2025ZD030) and Tianjin Key Medical Discipline Construction (TJYXZDXK‐3‐006B).

## Disclosures

The authors has nothing to report.

## Ethics Statement

All study protocols for animal experiments were approved by the Institutional Animal Care and Use Committee of Tianjin Medical University and conformed to the current NIH guidelines for Care and Use of Laboratory Animals.

## Conflicts of Interest

The authors declare no conflicts of interest.

## Supporting information




**Supporting File 1**: advs77379‐sup‐0001‐SuppMat.docx.

**Supporting File 2**: advs77379‐sup‐0002‐SuppMat.xlsx.

## Data Availability

The data that supports the findings of this study are available in the supplementary material of this article.
