# Supplementary material for: SerpinA3 is an Endogenous TGF‐β Receptor Antagonist that Attenuates Cardiac Fibroblast Activation and Fibrotic Remodeling
Source: Adv Sci (Weinh). 2026 Aug 30:e77379. Online ahead of print. doi: 10.1002/advs.77379 (PMC13526419; doi:10.1002/advs.77379)
Supplement: Supplementary file 1 [file ADVS-9999-e77379-s002.docx]

**Supplemental Figure 1** C57BL/6J mice underwent TAC surgery for the 8 weeks, the control group underwent sham surgery. **(A-G)** Echocardiographic analysis of left ventricular ejection fraction (EF), fractional shortening (FS), interventricular septal thickness at diastole (IVSTd), left ventricular posterior wall thickness at diastole (LVPWd), left ventricular internal diameter at end-diastole (LVIDd), left ventricular internal diameter at end-systole (LVIDs), left ventricular mass (LV mass) of 2 groups mice. Data are mean ± SEM, n = 6 mice per group, unpaired 2-tail t test. **(H)** Principal component analysis (PCA) for proteomic. **(I)** Differential expression of Serpins was compared between the 2 groups. **(J)** Representative confocal microscopy images of immunofluorescence staining for SerpinA3K (red) and vimentin (VIME), α-SMA, CD31 or α-actinin (green). Scale bar: 20 μm. **(K)** Quantification of SerpinA3K co-localization with vimentin-, α-SMA-, and CD31-positive cells shown in (J).

**Supplemental Figure 2** C57BL/6J mice underwent Sham or TAC surgery, followed by intraperitoneal injection of recombinant SerpinA3 protein (100ug/kg) every 3 days for 8 weeks. The control group was injected with sterile saline. **(A-E)** Echocardiographic analysis of interventricular septal thickness at diastole (IVSTd), left ventricular posterior wall thickness at diastole (LVPWd), left ventricular internal diameter at end-diastole (LVIDd), left ventricular internal diameter at end-systole (LVIDs), left ventricular mass (LV mass) in each group. Data are mean ± SEM, n = 6 mice per group, 2-way ANOVA with Bonferroni post-test. **(F)** Kaplan–Meier survival curves of saline- and SerpinA3-treated mice following TAC. **(G)** Violin plot showing detected genes per cell (left panel), numbers of unique molecular identifiers per cell (mid panel), and mitochondrial percent (right panel) after filter in each sample. **(H)** PCA was performed on single-cell transcriptomic data to visualize cell distribution along the first two principal components (PC1 and PC2).

**Supplemental Figure 3 (A)** Uniform manifold approximation and projection (UMAP) of cells colored by clusters in Saline and SerpinA3. 22 clusters indicated by colors are marked with the presumed cell types. **(B)** Heatmap showing the top markers per cluster in the scRNA-seq data. **(C)** Bar plot of the percentage of per cluster contributions in each scRNA-seq sample. **(D)** Pseudo-time analysis of CF subsets from Saline and SerpinA3.

**Supplemental Figure 4 (A-B)** C57BL/6J mice underwent Sham or TAC surgery, followed by intraperitoneal injection of recombinant SerpinA3 protein (100 μg/kg) every 3 days for 8 weeks. **(A)** mRNA levels of Col1a1, POSTN and CCN2 in the left ventricle of mice detected by RT-qPCR. **(B)** Quantification of t-Smad2/3 in Figure 4C. Data are mean ± SEM, n = 6 per group, 2-way ANOVA with Bonferroni post-test. **(C)** Mouse primary CFs (mPCFs) were pretreated with different concentrations of recombinant SerpinA3 protein for 30 minutes, followed by 2 hours of TGF-β1 (10ng/mL) stimulation. Quantification of protein levels of t-Smad2/3 detected by western blot analysis. n = 5 independent experiment. Data are mean ± SEM, 1-way ANOVA with Bonferroni post-test. **(D)** mPCFs were pretreated with recombinant SerpinA3 protein (100ng/μL) for 30 minutes, followed by 48 hours of TGF-β1 (10ng/mL) stimulation. mRNA levels of Col1a1, POSTN and CCN2 in mPCFs detected by RT-qPCR. **(E)** Representative images of cell monolayers at 0 h and 24 h after scratching. Scale bar: 200 μm. **(F)** Quantitative analysis of wound closure rate at different time points. n = 6 independent experiment. Data are mean ± SEM, 2-way ANOVA with Bonferroni post-test.

**Supplemental Figure 5** C57BL/6J mice were injected with AAV9-*postn*-Ctrl and AAV9-*postn*-SerpinA3 via the tail vein and underwent Sham or TAC surgery for 8 weeks. **(A-E)** Echocardiographic analysis of interventricular septal thickness at diastole (IVSTd), left ventricular posterior wall thickness at diastole (LVPWd), left ventricular internal diameter at end-diastole (LVIDd), left ventricular internal diameter at end-systole (LVIDs), left ventricular mass (LV mass) in each group. **(F)** mRNA levels of Col1a1, POSTN and CCN2 in the left ventricle of mice detected by RT-qPCR. **(G)** Quantification of protein levels of t-Smad2/3 was detected by western blot analysis. Data are mean ± SEM, n = 6 mice per group, 2-way ANOVA with Bonferroni post-test.
